# Immunostaining with D2–40 improves evaluation of lymphovascular invasion, but may not predict sentinel lymph node status in early breast cancer

**DOI:** 10.1186/1471-2407-9-109

**Published:** 2009-04-08

**Authors:** Anna V Britto, André A Schenka, Natália G Moraes-Schenka, Marcelo Alvarenga, Júlia Y Shinzato, José Vassallo, Laura S Ward

**Affiliations:** 1 Molecular Genetics of Cancer Laboratory, Department of Internal Medicine, School of Medical Sciences, State University of Campinas (Unicamp), São Paulo, Brazil; 2 Laboratory of Investigative and Molecular Pathology (CIPED), School of Medical Sciences, State University of Campinas (Unicamp), São Paulo, Brazil; 3 Department of Obstetrics and Gynecology, School of Medical Sciences State University of Campinas (Unicamp), São Paulo, Brazil

## Abstract

**Background:**

Sentinel lymph node (SLN) biopsy is a widely used diagnostic procedure in the management of early breast cancer. When SLN is free of metastasis, complete axillary dissection may be skipped for staging in clinically N0 patients, allowing a more conservative procedure. Histological tumor features that could reliably predict SLN status have not yet been established. Since the degree of tumor lymphangiogenesis and vascularization may theoretically be related to the risk of lymph node metastasis, we sought to evaluate the relationship between lymph vessel invasion (LVI), lymphatic microvascular density (LVD), microvascular density (MVD) and VEGF-A expression, with SLN status and other known adverse clinical risk factors.

**Methods:**

Protein expression of D2–40, CD34, and VEGF-A was assessed by immunohistochemistry on paraffin-embedded sections of primary breast cancer specimens from 92 patients submitted to SLN investigation. The presence of LVI, the highest number of micro vessels stained for D2–40 and CD34, and the protein expression of VEGF-A were compared to SLN status, clinicopathological features and risk groups.

**Results:**

LVI was detected in higher ratios by immunostaining with D2–40 (p < 0.0001), what would have changed the risk category from low to intermediate in four cases (4.3%). There was no association between LVI and other angiogenic parameters determined by immunohistochemistry with SLN macrometastases, clinical features or risk categories.

**Conclusion:**

Assessment of LVI in breast carcinoma may be significantly increased by immunostaining with D2–40, but the clinical relevance of altering the risk category using this parameter may not be advocated according to our results, neither can the use of LVI and LVD as predictors of SLN macrometastasis in early breast cancer.

## Background

Breast cancer is the most frequent neoplasm in women in several countries, including Brazil. In the last decades, detection of disease in earlier clinical stages has improved prognosis, however five-year disease-free survival of breast cancer presenting with T1 to 3, N0–1, and M0 staging remains at about 72% [1]. For this reason, continuing efforts to establish reliable prognostic markers are made.

Malignant neoplasms are angiogenesis-dependent [2]. The prognostic value of the tumor microvascular density (MVD) in breast cancer has been examined in several studies, with correlations with tumor recurrence, disease-free or overall survival [3-14]. Some discrepancies detectable on the subject may be explained by differences in patients' selection (age, menopausal status, tumor type, tumor size, adjuvant treatment, follow-up interval, number of patients included in each study), and in material and methods used (antibody's specificity, methods used to assess MVD). Considering meta-analysis results, significant evidence for the prognostic value of MVD in breast cancer was detected, however it was weak [15]. Vascular invasion around the tumor has been also considered an adverse risk factor in node-negative breast carcinoma [16]. It has been recently shown that assessing lymphovascular invasion (LVI) is related with other features of aggressiveness of breast cancer, as high proliferation index and low hormonal receptor [17]. In spite of the number of publications on the subject, the value of LVI and other angiogenesis markers in early breast carcinoma has not been sufficiently explored in part because the lack of reliable antibodies. The use of D2–40 in tumor pathology to detect lymphatic vessels is growing, but some controversy is expected because the experience in its use is quite recent. In the present study it was our purpose to evaluate the feasibility of vascular invasion assessed by hematoxilin-eosin (H&E) and by immunostaining with D2–40, as well as LVD, MVD and VEGF-A expression in early breast carcinoma, and its correlation to sentinel lymph node (SLN) status and to other clinicopathological parameters.

## Methods

Consecutive female patients diagnosed with early breast carcinoma and submitted to SLN examination were selected from 2004 to 2006 at the Women's Hospital of the State University of Campinas Medical School, Unicamp, São Paulo, Brazil. The present study was performed with the approval of the Committee of Ethics in Research of our institution, and was carried out in compliance with the Helsinki Declaration. Ninety-two cases were morphologically reanalyzed, both tumor and SLN. Medical records were revised; patient's age, tumor size, axillary lymph node involvement, risk group, pathological staging of tumor and lymph nodes and clinical evaluation for metastasis (pT pN cM) were determined as markers of aggressiveness. Staging was based on the AJCC Cancer Staging Manual, 6th Edition (2002) [18]. Histologic grade was determined by the modified Nottingham classification, proposed by Elston & Ellis [19]. Risk groups were classified according to Goldhirsh et al (2005) [16], as follows:

### Low risk

Node negative AND all of the following features: pT ≤ 2 cm, AND histological grade 1, AND absence of peritumoral vascular invasion, AND HER2/*neu *gene neither overexpressed nor amplified, AND age ≥ 35 years.

### Intermediate risk

IF node negative AND at least one of the following features: pT ≥ 2 cm, OR histological grade 2–3, OR presence of peritumoral vascular invasion, OR HER2/*neu *gene overexpressed or amplified, OR age < 35 years. OR IF less than four nodes are positive, AND HER2/*neu *gene neither overexpressed nor amplified.

### High risk

One to three nodes positive AND Her2/*neu *gene overexpressed or amplified, OR four or more nodes positive.

#### Sentinel Node (SLN)

SLNs were detected using a combination of radioactive colloid and Patent blue V. Three hours before surgery, 0.2 ml radioactive tracer (dextran mCi ^99 m^Tc-labelled) was injected subdermally at the tumor site. Twenty to 30 minutes afterwards, lymphatic scintillography was performed to localize SLNs. The skin overlying the node was marked with permanent ink. During the surgery, a blue dye (2 ml, 50 mg/ml) was applied at the lesion site by a subdermal injection. SLNs were then detected simultaneously by a hand-held gamma ray detecting probe and visual control. All surgically removed SLNs underwent intraoperative cyto- (imprints) and histopathological examination. Gross processing of the nodes consisted of serial sectioning in 1–2 mm thick slices, which were fixed in 10% formalin and embedded in paraffin. They were then microscopically evaluated in serial H&E stained sections at intervals of 150 μm [20]. In negative cases, two slide samples were additionally stained with an anti-pancytokeratin antibody (AE1/AE3, Dako, Carpenteria, CA, USA; 1:200). As the clinical value of isolated neoplastic cells and the presence of micrometastasis (0.2 – 2 mm) is still controversial for decision taking, in the present study SLN were considered positive for statistical analysis only when they contained tumor foci greater than 2 mm (macrometastasis).

#### Immunohistochemistry

Briefly, two levels of 4-μm thick sections were obtained, placed on silanized slides, deparaffinized in xylene and rehydrated. Endogenous peroxidase activity was quenched with 3% hydrogen peroxide. Antigen retrieval was performed by heating slides in citrate buffer (10 mM, pH 6.0) at 95°C for 30 minutes. The primary monoclonal antibodies used were mouse anti-human VEGF-A (CloneVG1, Dako, diluted at 1:100), CD34-Class II (Clone QBend-10, Dako, 1:50), D2–40 (Clone D2–40, Dako, 1:50), estrogen receptor (Clone 1D5, Dako, 1:150), progesterone receptor (Clone PGR636, Dako, 1:300), and HER-2/*neu *(Clone PN2A, Dako, 1:450). The Max Polymer Detection System (Novolink, Novocastra, Newcastle, UK) was used to reveal antigen-antibody reaction. Staining was achieved using 3,3-diamonobenzidine tetrahydrochlride (Sigma, St. Louis, MO, USA) and counterstaining using Mayer's hematoxilin. All reactions were performed using appropriate positive and negative controls.

All scores are represented by the mean of the two levels evaluated by immunohistochemistry. Immunostaining for hormone receptors were scored as negative or positive (0 and 2 *vs *3 to 8, according to Harvey et al, 1999) [21]. Immunostaining for HER-2/*neu *was scored as negative, 1+ to 3+, according to previously described parameters [22]. Cases scored as 2+ were additionally submitted to chromogenic "in situ" hybridization (CISH, Zymed's SpoT-Light^R ^HER2CISH™, Invitrogen, San Francisco, CA, USA), according to the manufacturer's instructions [23]. VEGF-A expression was scored based on extent or percentage of positive cells (0: negative; 1: <25%; 2: 26–50%; 3; 51–75%; 4: > 75%) and staining intensity (0: negative; 1: weak; 2: moderate; 3: strong). The sum of both parameters resulted in a final VEGF-A score, which was then grouped into three categories: negative to weak (0–2), moderate (3–5) and strong (6–7) immunostaining.

#### Microvessel density and vascular invasion assessment

Blood and lymphovascular microvessel density were assessed independently by two observers, without knowledge of the patients' clinicopathologic data. Immunostained tumor sections were scanned at low magnification (40×) and the three most densely vascular areas ("hot spots") were photographed at magnification 200× (0.74 mm^2 ^per field). Vessels stained by CD34 or D2–40 were counted using an image analysis software (Imagelab^®^, version 2.4, São Paulo, Brazil). Tumor lymphatic vessel invasion was established when at least one neoplastic cell cluster was clearly visible inside a D2–40 positive lymph vessel according to Yamauchi et al, 2006 [24]. Tumor lymphatic invasion thus determined was compared with the one independently reported in H&E stains.

### Statistical analyses

Statistical analysis was performed using the SAS System for Windows software (version 9.1.3). Associations were assessed using 2 × 2 or 2 × n contingency table analysis. The Fisher's exact test was used to examine relationship between VEGF-A expression and risk groups, pathological stage and SLN status, as well as clinicopathological features (tumor size, menopausal status, histological type, histological grade, pathological stage, estrogen and progesterone receptors, Her2/*neu *expression, vascular invasion on HE and on D2–40 stained slides) and sentinel node status. Multiple logistic regression analysis was used to evaluate the effect of microvascular density and vascular invasion markers, after adjusting for other potential confounders such as age and tumor size. Correlations between CD34 and D2–40 expression levels, as well as between these antibodies and VEGF-A expression groups were calculated using the linear Spearman's coefficient. Vascular invasion on H&E and D2–40 staining were analyzed using the McNemar test. The Kappa value was calculated to assess inter-observer variability concerning VEGF-A expression. The interclass correlation coefficient was determined to evaluate inter-observer variability concerning the expression of CD34 and D2–40. The significance level was set at p = 0.05 for all tests.

## Results

Median age of patients was 55 y (range 32–77 y). Twenty-three patients were pre-menopausal and 69 menopausal. Stage of disease was I in 58 patients, II in 26 and III in 8. Tumor size presented a median of 12 mm (range 2–50 mm). Eighty cases corresponded to the ductal type, 5 were lobular and 7 of other types. Histological grade was I in 36 patients, II in 47 and III in 9.

SLN was positive in 31 patients (33.7%), more frequently in pre-menopausal women (52.1% *vs*. 27.5%; p = 0.03). Patients with positive SLN were significantly younger: median 53 y (range 33–68 y) *vs *57 y (range 32–77 y; p = 0.013). Six patients were included in the low risk group (6.5%), 73 in the intermediate risk group (79.3%) and 13 in the high risk group (14.2%). Estrogen receptor status was positive in 77 patients and negative in 14. Progesterone receptor was positive in 76 patients and negative in 15. HER2/neu status was negative in 61 patients and positive (either 3+ by immunohistochemistry or amplified by CISH) in 29. In one patient it was not possible to assess ER and PR status; the same occurred in two patients related to HER2/neu status. (Lymph-)vascular invasion (LVI) was present in 6.5% (six patients) of the cases on H&E slides and in 29.3% (27 patients) on D2–40 stained sections. The difference between both methods of evaluation was significant (McNemar test: p < 0.0001). When both methods of evaluation of LVI were compared to each other, an agreement ratio of 75% was found (Table 1). When both methods of evaluation of LVI were compared to SLN status, either grouping the number of positive SLN in 0 *vs *≥ 1 (Table 2), or 0 and up to 3 *vs *≥ 4 (Table 3), no significant correlation was found. When both methods of evaluation of LVI were compared to the three risk groups (Table 4), only evaluation by H&E showed significant correlation to risk groups (p = 0.04).

**Table 1:** Correlation between evaluation of (lympho-)vascular invasion assessed by hematoxilin & eosin (H&E) staining and by immunohistochemistry using the D2–40 antibody

Vascular invasion assessed by H&E	Lymph vascular invasion assessed by immunostaining with D2–40		Total
		
	*Negative*	*Positive*	
*Negative*	64	22	86
*Positive*	1	5	6

**Total**	65	27	92

75% agreement; there was no relation between both methods by the Fisher's exact test (*p *= 0.335); accordingly, the difference between both methods of assessing vascular invasion was significantly different by the McNemar test (*p *< 0.0001)

**Table 2:** Correlation between (lympho-)vascular invasion and sentinel lymph node (SLN) status

SLN	Vascular invasion assessed by H&E		Lymph vascular invasion assessed by immunostaining with D2–40	
	
	*Negative*	*Positive*	*Negative*	*Positive*
*Negative*	57	4	44	17
*Positive*	29	2	21	10
**Total**	86	6	65	27

*p *values*	0.090		0.376	

* Fisher's exact test

**Table 3:** Correlation between four or more positive and negative + up to three sentinel lymph nodes (SLN) and status of (lympho-)vascular invasion

SLN	Vascular invasion assessed by H&E		Lymph vascular invasion assessed by immunostaining with D2–40	
	
	*Negative*	*Positive*	*Negative*	*Positive*
*Negative or <4 positive*	80	6	60	26
≥ 4 *positive*	6	0	5	1
**Total**	86	6	65	27

*p *values*	0.103		0.543	

* Fisher's exact test

**Table 4:** Correlation between the three risk groups and status of (lympho-) vascular invasion

Risk groups^a^	Vascular invasion assessed by H&E		Lymph vascular invasion assessed by immunostaining with D2–40	
	
	*Negative*	*Positive*	*Negative*	*Positive*
*Low*	24	1	15	9
*Intermediate*	49	5	41	15
*High*	13	0	9	3
**Total**	86	6	65	27

*p *values*	0.040		0.221	

* Fisher's exact test; a: according to Goldhirsh et al, 2005 [ref. # [16]]

When patients' data were analyzed according to the presence (n = 28) and absence (n = 64) of LVI by either method, H&E or D2–40, no correlation was found according to patients' age, tumor size, histological type or grade, MVD counts (CD34), LVD (D2–40), VEGF-A, HER-2/*neu *and hormonal receptor status, or to SLN status.

Immunostaining with D2–40 would have resulted in upgrade of risk group classification of four cases (4.3%). Multiple logistic regression revealed a relationship between SLN status and tumor size (p = 0.0012) and age (p = 0.013). There was no relationship between SLN status and all other clinicopathological variables: stage of the disease, hormonal receptor and HER2/*neu *status, vascular invasion (assessed by H&E or D2–40), VEGF-A immunostaining, MVD, or LVD (Table 5), after adjusting for tumor size and age. There was no correlation between risk groups, LVD, MVD or VEGF-A expression. Spearman's coefficient between CD34, D2–40 and VEGF-A was low, reflecting lack of significant correlation. Her2/*neu *was not correlated to VEGF-A, CD34 and D2–40. The interclass correlation coefficient between two observers related to CD34 and D2–40 were, respectively, 0.995 (CI95% = 0.989–0.997) and 0.87 (CI95% = 0.63–0.90). Kappa value was 0.7154 (CI95% = 0.48–0.95).

**Table 5:** Correlation between sentinel lymph node (SLN) status and microvascular density (MVD), lymphovascular density (LVD) and intensity of VEGF-A

*Marker*		*All patients*	*SLN +*	*SLN -*	*p *values*
**VEGF-A^a^**	Negative and weakly positive	37	13	24	0.32
	Moderately positive	25	12	13	
	Strongly positive	30	6	24	
**MVD (CD34)^b^**		79 (24–162)	79 (30–160)	81(24–162)	0.78
**LVD (D2–40)^b^**		7 (0–22)	8 (0–22)	7 (1–20)	0.73

* adjusted for tumor size and age; a: values correspond to the number of patients in each group; b: values correspond to median vessel count; range is in parenthesis.

## Discussion

The presence of lymph node metastasis is one of the most important prognostic factors in breast cancer [25]. Our data showed that only younger age, pre-menopausal status (both parameters reflecting approximately the same biological phenomenon), and larger tumor size were related to SLN status, which is consistent with the known adverse influence of these parameters in defining risk groups, particularly in early breast cancer [26]. The present data support the value of performing immunostaining using the antibody D2–40 to significantly improve evaluation of LVI. This finding is in keeping with most studies which performed the comparison between assessments of LVI by H&E and D2–40 stains [17,24,27-30]. However, as neither method significantly improved predictability of SLN status in the current study, it is yet not possible to sustain the habitual use of D2–40 staining in the evaluation of risk categories.

In four of our cases (4.3%), the immunohistochemical assessment of LVI would have changed the risk group of the patients from low to intermediate. However, the relevance of this upgrade for the management of the patient cannot be assured. It has been evidenced elsewhere that the presence of vascular invasion was significantly associated with poorer disease free survival both in the entire group of patients analyzed, and when only the group presenting positive non-sentinel lymph node was considered. However, only the results obtained with D2–40, but not on H&E stain, were statistically significant [29]. The opposite has been stated in another study, in which the authors referred a concordance of 78.2% between LVI in H&E and with D2–40 (similar to ours, 75%). Only the values obtained on H&E stained sections were correlated with positive SLN [31]. In a recent report, when LVI was correlated with the presence of micro and macrometastases in axillary lymph nodes, only the evaluation on H&E stains was significantly associated with the presence of macrometastasis in SLN [17]. These differences raise the issue that the increase in sensitivity in LVI detection by immunohistochemistry might not be associated with clinically relevant impact, meaning that only higher degrees of vascular invasion easily detected on H&E stains are important. The practical value of utilizing the presence of LVI to predict SLN status could also be questioned, first because SLN evaluation does not imply a major surgical risk for the patient. In addition, most cases are diagnosed in a small sample of the tumor, and the absence of LVI in the original slides would not preclude SLN evaluation [32]. Also, even in those studies which demonstrate correlation between the presence of LVI and lymph node status present a percentage of discordance, a fact that will make it advisable to stick to the actual standardized procedures. The significance shown herein between vascular invasion assessed by H&E and risk categories is borderline and might not correspond to a consistent biologic fact, as all of the 13 cases placed in the high risk group showed negative SLN.

Another issue evidenced by the present findings is the contradiction of the expected SLN colonization by tumor cells, as they should be transported by the invaded peritumoral lymphatic vessels. A parallel verification could help to explain this finding: neoplastic involvement of non-SLN is not invariably present in patients with positive SLN [33]. Then, it could be argued that the simple presence of foci of neoplastic cells in more proximal regions of the lymphatic flow does not necessarily imply in their capability of escaping defense mechanisms, which may prevent their spread to the next step.

Assessment of vascular invasion in breast cancer is not taken into account in a widely used prognostic index, but it was included as a parameter in grouping patients with different risks, using H&E stains [16,32]. Considering the above data, it might be important to reevaluate the reliability of including the assessment of LVI by immunohistochemistry using the antibody D2–40 in the prognostic index in large international trials, before using this method individually, which could be hazardous for the patient by overestimating the risk category. Such procedure is technically viable and would allow the comparison of data from reports in which only conventionally stained slides to detect vascular invasion have been used [34-36]. For the sake of consistency, the topography in which LVI is searched should be considered the border of neoplasia, as data on the lymphangiogenesis in inner areas of the tumor and its possible relevance for prognosis is a matter of debate, perhaps because of differences in the markers used [24,31,37]. Quantification of vascular invasion around the tumor should also be evaluated, as this parameter could reflect more properly the likelihood of dissemination of the disease, as suggested before [36]. In other words, only pronounced vascular infiltration, rather than focal or sparse, could more likely suggest that tumor cells have acquired the potential to nest at distance.

For this purpose, D2–40 seems a good marker for lymphatic vessels, in spite of the possible pitfalls in interpretation of its reactivity. Rabban & Chan (2008) suggested that p63 should be simultaneously used to stain myoepithelial cells when it renders difficult to distinguish between lymphangiovascular invasion and stromal myoepithelial cells in close relation to neoplastic cells [38]. Arnaout-Alkarain et al (2007) have used anti-CD31 to assist decision taking in cases with discordant results between LVI assessment with D2–40 and H&E [27]. It has been also suggested that double staining using D2–40 in conjunction with proliferation marker Ki-67 should be preferred to avoid misinterpretation of LVI [39]. It should be kept in mind that the opposite can occur. In cases of minimal vascular invasion on H&E stains, further sections obtained for immunohistochemistry may not represent the same area of vascular invasion, resulting negative [17].

Our data demonstrated no association between MVD, LVD, and VEGF-A protein expression with SLN status and other clinicopathological features. Lack of relationship between markers of angiogenesis and clinicopathological features has been observed in other studies, but most reports have shown prognostic value of increased MVD and/or presence of LVI [12,24,28-31,40-44]. In additional studies, however, multivariate analysis has eliminated the prognostic value of angiogenic parameters [27,37]. The reason for the discrepancy between our results and other reports seems not to be related to selection bias, as we have studied a well defined group of patients, using the same clinical management protocol. We also used sensitive markers and well established techniques to determine MVD and LVD [45-47]. Moreover, the concordance rate between the two observers was satisfactory. However, unexpected drawbacks, such as inadequacy of antigen preservation, or differences due to dissimilar sources of antibodies might have accounted for this divergence.

The lack of correlation between LVD and SLN status also seems in opposition to the reasonable concept that the more vessels are around a tumor, the more likely it is to metastasize. However, in this respect we are dealing with the complex mechanisms of circulation and nesting of neoplastic cells, which might not be properly evaluated by the static methods used herein.

## Conclusion

The data presented herein support the value of assessing LVI by immunohistochemistry using D2–40, in increasing the ratio of positive cases. It may be suggested that this parameter should be included in the evaluation of risk groups in patients with early breast cancer, in order to add a more reproducible parameter to compare different trials, and to determine in which extent this enhancement in sensitivity of the method will reveal clinical relevance. For now, the evidences from our study do not allow us to advocate the use of LVI and MVD or LVD as predictors of SLN invasion in breast cancer.

## Abbreviations

CD: cluster of differentiation; CISH: chromogenic *in situ *hybridization; H&E: hematoxilin and eosin; LVD: lymphatic microvascular density; LVI: lymph vessel invasion; MVD: microvascular density; SLN: sentinel lymph node; VEGF-A: vascular endothelial growth factor.

## Competing interests

The authors declare that they have no competing interests.

## Authors' contributions

AVB and LSW designed and participated in all steps of the study. AAS and JV participated in the analysis of immunohistochemical data, in statistical analysis and discussion of the results. NGMS and MA reviewed all histopathological slides. JYS proceeded surgeries and follow up of patients. All authors read and approved the final version manuscript.

## Pre-publication history

The pre-publication history for this paper can be accessed here:

http://www.biomedcentral.com/1471-2407/9/109/prepub

## References

[B1] Adjuvant Breast Cancer Trials Collaborative Group Polychemotherapy for early breast cancer: results from the International Adjuvant Breast cancer Chemotherapy Randomized Trial J Natl Cancer Inst 2007 99 506 515 17405995 10.1093/jnci/djk108

[B2] Gasparini G Clinical significance of determination of surrogate markers of angiogenesis in breast cancer Crit Rev Oncol Hematol 2001 37 97 114 11166583 10.1016/s1040-8428(00)00105-0

[B3] Weidner N Semple JP Welch WR Folkman J Tumor angiogenesis and metastasis-correlation in invasive breast cancer N Engl J Med 1991 324 1 8 1701519 10.1056/NEJM199101033240101

[B4] Ogawa Y Chung YS Nakata B Takatsuba S Maeda K Sawada T Kato Y Yoshikawa K Sakurai M Sowa M Microvessel quantification in invasive breast cancer by staining for factor VIII-related antigen Br J Cancer 1995 71 297 301 10.1038/bjc.1995.251 PMC2033832 7779727

[B5] Simpson JF Ahn C Battifora H Esteban JM Endothelial area as a prognostic indicator for invasive breast carcinoma Cancer 1996 77 2077 2085 8640673 10.1002/(SICI)1097-0142(19960515)77:10<2077::AID-CNCR17>3.0.CO;2-S

[B6] Martin L Green B Renshaw C Lowe D Rudland P Leinster SJ Winstanley J Examining the technique of angiogenesis assessment in invasive breast cancer Br J Cancer 1997 76 1046 1054 9376265 10.1038/bjc.1997.506 PMC2228092

[B7] Gasparini G Toi M Verderio P Ranieri G Dante S Bonoldi E Boracchi P Fanelli M Tominaga T Prognostic significance of p53, angiogenesis, and other conventional features in operable breast cancer: subanalysis in node-positive and node-negative patients Int J Oncol 1998 12 1117 11125 9538138 10.3892/ijo.12.5.1117

[B8] De Placido S Carlomagno C Ciardello F de Laurentiis M Pepe S Ruggiero A Tortora G Panico L D'Antonio A Pettinato G Petrella G Bianco AR Measurement of neovascularization is an independent prognosticator of survival in node-negative breast cancer patients with long-term follow-up Clin Cancer Res 1999 5 2854 2859 10537353

[B9] Kumar S Ghellal A Li C Byrne G Haboubi N Wang JM Bundred N Breast carcinoma:vascular density determined using CD105 antibody correlates with tumor prognosis Cancer Res 1999 59 856 61 10029075

[B10] Hansen S Grabau DA Sorensen FB Bak M Vach W Rose C Vascular grading of angiogenesis:prognostic significance in breast cancer Br J Cancer 2000 82 339 347 10646886 10.1054/bjoc.1999.0924 PMC2363269

[B11] Tsutsui S Kume M Era S Prognostic value of microvessel density in invasive ductal carcinoma of the breast Breast Cancer 2003 10 312 319 14634509 10.1007/BF02967651

[B12] Schoppmann FS Bayer G Aumayer K Taucher S Geleff S Rudas M Kubista E Hausmaninger H Samonigg H Gnant M Jakesz R Horvat R Austrian Breast and Colorectal Cancer Study Group Prognostic value of lymphangiogenesis and lymphovascular invasion in invasive breast cancer Annals Surg 2004 240 306 312 10.1097/01.sla.0000133355.48672.22 PMC1356408 15273556

[B13] Nakamura Y Yasuoka H Tsujimoto M Imabun S Nakahara M Nakao K Nakamura M Mori I Kakudo K Lymph vessel density correlates with nodal status, VEGF-A-C, expression and prognosis in breast cancer Breast Cancer Res Treat 2005 91 125 132 15868440 10.1007/s10549-004-5783-x

[B14] Mohammed RA Green A El-Shikh S Paish EC Ellis IO Martin SG Prognostic significance of vascular endothelial cell growth factors-A, C and D in breast cancer and their relationship with angio and lymphangiogenesis Br J Cancer 2007 96 1092 1100 17353919 10.1038/sj.bjc.6603678 PMC2360132

[B15] Uzzan B Nicolas P Cucherat M Perret GY Microvessel density as a prognostic factor in women with breast cancer: a systematic review of the literature and meta-analysis Cancer Res 2004 64 2941 2955 15126324 10.1158/0008-5472.can-03-1957

[B16] Goldhirsch A Glick JH Gelber RD Coates AS Thurlimann B Senn HJ Panel members Meeting highlights: international expert consensus on the primary therapy of early breast cancer 2005 Ann Oncol 2005 16 1569 1583 16148022 10.1093/annonc/mdi326

[B17] Marinho VF Metze K Sanches FS Rocha GF Gobbi H Lymph vascular invasion in invasive mammary carcinomas identified by the endothelial lymphatic marker D2–40 is associated with other indicators of poor prognosis BMC Cancer 2008 8 64 18307818 10.1186/1471-2407-8-64 PMC2294134

[B18] Greene FL Page DL Fleming ID Fritz AG Balch CM Haller DG Morrow M Greene FL, Page DL, Fleming ID, Fritz AG, Balch CM, Haller DG, Morrow M Breast AJCC Cancer Staging Manual 2002 6 New York: Springer 223 240

[B19] Elston CW Ellis IO Pathological prognostic factors in breast cancer. I. The value of histological grade in breast cancer: experience from a large study with long-term follow-up Histopathology 1991 19 403 410 1757079 10.1111/j.1365-2559.1991.tb00229.x

[B20] Cserni G Evaluation of sentinel lymph nodes in breast cancer Histopathology 2005 46 697 706 15910602 10.1111/j.1365-2559.2005.02143.x

[B21] Harvey JM Clark GM Osborne K Allred DC Estrogen receptor status by immunohistochemistry is superior to the ligand-binding assay for predicting response to adjuvant endocrine therapy in breast cancer J Clin Oncol 1999 17 1474 1481 10334533 10.1200/JCO.1999.17.5.1474

[B22] Yaziji H Taylor CR Begin at the beginning, with the tissue! The key message underlying the ASCO/CAP Task-force Guideline Recommendations for HER2 testing Appl Immunohistochem Mol Morphol 2007 15 239 241 17721265 10.1097/PAI.0b013e3181250254

[B23] Tanner M Gancberg D Di Leo A Larsimont D Rouas G Piccart MJ Isola J Chromogenic in situ Hybridization. A practical alternative for fluorescence in situ hibridization to detect HER-2/neu oncogene amplification in archival breast cancer samples Am J Pathol 2000 157 1467 72 11073807 10.1016/S0002-9440(10)64785-2 PMC1885742

[B24] Yamauchi C Hasebe T Iwasaki M Imoto S Wada N Fukayama M Ochiai A Accurate assessment of lymph vessel tumor emboli in invasive ductal carcinoma of the breast according to tumor areas, and their prognostic significance Hum Pathol 2007 38 247 259 17056095 10.1016/j.humpath.2006.07.017

[B25] Carter CL Allen C Henson DE Relation of tumor size, lymph node status and survival in 24,740 breast cancer cases Cancer 1989 63 181 187 2910416 10.1002/1097-0142(19890101)63:1<181::aid-cncr2820630129>3.0.co;2-h

[B26] Rivadeneira DE Simmons RM Christos PJ Hanna K Daly JM Osborne MP Predictive Factors associated with Axillary Lymph Node Metastases in T1a and T1b Breast Carcinomas: Analysis in More Than 900 Patients J Am Coll Surg 2000 191 1 6 10898177 10.1016/s1072-7515(00)00310-0

[B27] Arnaout-Alkarain A Kahn HJ Narod SA Sun PA Marks AN Significance of lymph vessel invasion identified by the endothelial lymphatic marker D2–40 in node negative breast cancer Mod Pathol 2007 20 183 191 17206106 10.1038/modpathol.3800728

[B28] Braun M Flucke U Debald M Walgenbach-Bruenagel G Walgenbach K-J Holler T Polcher M Wolfgarten M Sauerwald A Keyver-Paik M Kuehr M Buttner R Kuhn W Detection of lymphovascular invasion in early breast cancer by D2–40(podoplanin): a clinically useful predictor for axillary lymph node metastasis Breast Cancer Res Treat 2008 112 3 503 511 18165897 10.1007/s10549-007-9875-2

[B29] Tezuka K Onoda N Takashima T Takagaki K Ishikawa T Wakasa T Wakasa K Hirakawa K Prognostic significance of lymphovascular invasion diagnosed by lymphatic endothelium immunostaining in breast cancer patients Oncol Rep 2007 17 997 1003 17390035

[B30] El-Gohary YM Metwally G Saad RS Robinson MJ Mesko T Poppiti RJ Prognostic significance of intratumoral and peritumoral lymphatic density and blood vessel density in invasive breast carcinomas Am J Clin Pathol 2008 129 578 586 18343785 10.1309/2HGNJ1GU57JMBJAQ

[B31] Ito M Moriya T Ishida T Usami S Kasajima A Sasano H Ohuchi N Significance of Pathological Evaluation for Lymphatic Vessel Invasion in Invasive Breast Cancer Breast Cancer 2007 14 381 387 17986803 10.2325/jbcs.14.381

[B32] Chagpar AB Scoggins CR Martin RC 2nd Carlson DJ Laidley AL El-Eid SE McGlothin TQ Prediction of sentinel lymph node-only disease in women with invasive breast cancer Am J Surg 2006 192 882 887 17161113 10.1016/j.amjsurg.2006.08.063

[B33] Leidenius MHK Vironen JH Riihelä MS Krogerus LA Toivonen TS von Smitten KAJ Heikkilä PS The prevalence of non-sentinel node metastasis in breast cancer patients with sentinel node micrometastasis EJSO 2005 31 13 18 15642420 10.1016/j.ejso.2004.09.012

[B34] Choi SH Barsky SH Chang HR Clinicopathologic Analysis of Sentinel Lymph Node mapping in early breast cancer Breast J 2003 9 153 162 12752622 10.1046/j.1524-4741.2003.09304.x

[B35] Viale G Zurrida S Maiorano E Mazzarol G Pruneri G Paganelli G Maisonneuve P Veronesi U Predicting the status of axillary sentinel lymph nodes in 4351 patients with invasive breast carcinoma treated in a single institution Cancer 2005 103 492 500 15612028 10.1002/cncr.20809

[B36] Colleoni M Rotmensz N Maisonneuve P Sonzogni A Pruneri G Casadio C Luini A Veronesi P Intra M Galimberti V Torrisi R Andrighetto S Ghisini R Goldhirschi A Viale G Prognostic role of the extent of peritumoral vascular invasion in operable breast cancer Annals of Oncology 2007 18 1632 1640 17716986 10.1093/annonc/mdm268

[B37] Bono P Wasenius V-M Heikkila P Lundin J Jackson DG Joensuu H High LYVE-1-Positive Lymphatic Vessel Numbers Are Associated with Poor Outcome in Breast Cancer Clinical Cancer Research 2004 10 7144 7149 15534085 10.1158/1078-0432.CCR-03-0826

[B38] Rabban JT Chen YY D2–40 expression by breast myoepithelium:potential pitfalls in distinguishing intralymphatic carcinoma from in situ carcinoma Human Pathology 2008 39 175 183 18206495 10.1016/j.humpath.2007.06.018

[B39] Auwera I Van der Cao Y Tille JC Pepper MS Jackson DG Fox SB Harris AL Dirix LY Vermeulen PB First international consensus on the methodology of lymphangiogenesis quantification in solid human tumours Br J Cancer 2006 95 1611 1625 17117184 10.1038/sj.bjc.6603445 PMC2360768

[B40] Gasparini G Prognostic value of vascular endothelial growth factor in breast cancer Oncologist 2000 5 Suppl 1 37 44 10804090 10.1634/theoncologist.5-suppl_1-37

[B41] Fridman V Humblet C Bonjean K Boniver J Assessment of tumor angiogenesis in invasive breast carcinomas: absence of correlation with prognosis and pathological factors Virchows Arch 2000 437 611 617 11193472 10.1007/s004280000292

[B42] Ludovini V Sidoni A Pistola L Bellezza G de Angelis V Gori S Mosconi AM Bisagni G Cherubini R Bian AR Rodinò C Sabbatini R Mazzocchi B Bucciarelli E Tonato M Colozza M Evaluation of the prognostic role of vascular endothelial growth factor and microvessel density in stages I and II breast cancer patients Breast Cancer Res Treat 2003 81 159 168 14572158 10.1023/a:1025755717912

[B43] Nicolini A Campani D Miccoli P Spinelli C Carpi A Menicagli M Ferrari P Gadducci G Rossi G Fini M Giavaresi G Bonazzi V Giardino R Vascular endothelial growth factor (VEGF-A) and other common tissue prognostic indicators in breast cancer: a case-control study Int J Biol Markers 2004 19 275 281 15646833 10.1177/172460080401900404

[B44] Mylona E Alexandrou P Mpakali A Giannopoulou I Liapis G Markaki S Keramopoulos A Nakopoulou L The prognostic value of vascular endothelial growth factors (VEGF-As)-A and B and their receptor, VEGF-AR-1, in invasive breast carcinoma Gynecol Oncol 2007 104 557 563 17150246 10.1016/j.ygyno.2006.09.031

[B45] Evangelou E Kyzas PA Trikalinos TA Comparison of the diagnostic accuracy of lymphatic endothelial markers: Bayesian approach Mod Pathol 2005 18 1490 1497 15990898 10.1038/modpathol.3800457

[B46] Vermeulen PB Gasparini G Fox SB Fox SB Colpaert C Marson LP Gion M Belien JA de Waal RM Van Marck E Magnani E Weidner N Harris AL Dirix LY Second international consensus on the methodology and criteria of evaluation of angiogenesis quantification in solid tumours Eur J Cancer 2002 38 1564 1579 12142044 10.1016/s0959-8049(02)00094-1

[B47] Weidner N Current pathologic methods for measuring intratumoral microvessel density within breast carcinoma and other solid tumors Breast Cancer Res Treat 1995 36 169 180 8534865 10.1007/BF00666038

